# High-risk factors for subjective discomfort due to lower limb discrepancy after medial open wedge high tibial osteotomy

**DOI:** 10.1186/s13018-021-02542-y

**Published:** 2021-07-07

**Authors:** Axiang He, Yin Wang, Yanan Chen, Ying Zhou, Hui Zhang, Yanjie Mao, Wanjun Liu, Xianlong Zhang

**Affiliations:** 1 Department of Microbiology, Shanghai Ocean University, Shanghai, China; 2 Department of Orthopedics, Shanghai Sixth People’s Hospital, Shanghai Jiaotong University, Shanghai, China; 3 Department of Anesthesiology, Shanghai Sixth People’s Hospital, Shanghai Jiaotong University, Shanghai, China

**Keywords:** High tibial osteotomy, Lower limb discrepancy, High-risk factors

## Abstract

**Background:**

Medial open wedge high tibial osteotomy (OWHTO) may result in lower limb discrepancy (LLD), and some patients experience uncomfortable symptoms. Studies have found that the degree of LLD is one but not the only high-risk factor for inducing symptoms. The main purpose of this study is to explore the risk factors for symptomatic LLD.

**Methods:**

Sixty-four patients who underwent OWHTO in our hospital between June 2018 and January 2020 were included in the study. Changes in tibia length, lower limb length, femorotibial angle (FTA), LLD, and KOOS score were evaluated before and 1 year after surgery. Questionnaire was used to investigate whether patients had uncomfortable symptoms of LLD after surgery. Binary logistic regression was applied to analyze the risk factors of symptomatic LLD.

**Results:**

There were 18 patients with subjective LLD uncomfortableness, 13 of them were occasional and 5 were frequent. Patients had a mean correction angle of 11.7° ± 4.6°, with a mean increase in tibial length of 6.0 ± 3.5 mm, lower limb length of 7.5 ± 2.3 mm, and LLD of 6.9 ± 4.2 mm at 1 year post-operation. Preoperative and postoperative changes in tibia length and lower limb length were statistically significant (*P* < 0.0001).There were statistically significant differences in pain, symptoms, ADL, sports/recreations, QOL of KOOS subclassification before and after surgery (*P* < 0.0001). Binary logistic regression revealed that age ≥ 55, BMI ≥ 28, and LLD ≥ 10 mm were high-risk factors for symptomatic LLD (*P* = 0.031, OR = 4.82; *P* = 0.012, OR = 6.251; *P* = 0.006, OR = 6.836).

**Conclusion:**

Patients with age ≥ 55, BMI ≥ 28, and postoperative LLD ≥ 10 mm are more likely to develop symptomatic LLD. Older or heavier patients, who are expected to have an LLD greater than 10 mm after OWHTO should be fully informed of the possibility of postoperative LLD symptoms.

## Background

As a classic knee preserving surgery, high tibial osteotomy (HTO) has been developed for nearly 60 years and is one of the effective surgical interventions for treating medial compartment osteoarthritis of the knee combined with varus deformity [1]. After varus deformity of lower limbs, the stress concentration in the medial compartment during loading increases the pressure on the surface of the cartilage, and when it exceeds the bearing capacity, the articular cartilage injuries can occur, leading to osteoarthritis, resulting in knee pain and limited activity, which affect the patient’s quality of life. Meanwhile, due to the continuous increase of pressure in the medial compartment, the medial joint space is gradually narrowed, which easily leads to relaxation of the lateral collateral ligament (LCL) and contraction of the medial collateral ligament (MCL), thus further aggravating the varus deformity and osteoarthritis, forming a vicious circle [2, 3]. By correcting the mechanical axis of the lower limbs, HTO shifts the weight-bearing line outward, decreases the stress concentration of the medial compartment, thus effectively blocks the vicious cycle and delays the progression of OA [4].

The correction of lower limb weight bearing line can be achieved by either medial open wedge high tibial osteotomy (OWHTO) or lateral closed wedge high tibial osteotomy (CWHTO) [5]. At present, many studies have confirmed that both of the two methods can effectively make the weight-bearing line move out, so as to alleviate knee pain and delay the progress of OA. Although CWHTO has a certain risk of common peroneal nerve injury, no one is better than the other [4, 6]. Changes in limb length due to CWHTO are usually negligible, whereas OWHTO lengthens the lower limb on the operative side, resulting in high incidence of LLD. Studies have suggested that lower back pain and gait abnormalities are at risk when 5–10 mm of LLD are present; however, not all patients with LLD more than 10 mm are symptomatic. Furthermore, a proportion of patients with LLD < 5 mm still have subjective sensation regarding the lower limb length discrepancy [7, 8]. So, the degree of LLD is not the only factor that determines whether or not there are symptomatic. Through screening, we hypothesized that age, gender, body mass index (BMI), and the degree of LLD were the high-risk factors for symptomatic LLD after OWHTO. The purpose of this study was to explore the risk factors for symptomatic LLD, so as to assist clinical decision-making and improve the level of treatment.

## Materials and methods

### Patient data

Sixty-four patients who underwent OWHTO in our hospital between June 2018 and January 2020 were included in the study. Inclusion criteria were (1) be diagnosed with varus deformity of the lower limb with osteoarthritis of the medial compartment, K-L grade II–III, no bone-to-bone wear or full-thickness cartilage injury. (2) No or mild osteoarthritis in the contralateral tibial-femoral joint and patellofemoral joint. (3) Varus deformity ranged from 5° to 20°, medial proximal tibia angle (MPTA) < 85°. (4) The range of motion was more than 90°, flexion contracture was less than 10°, and there was no knee instability. Exclusion criteria were (1) combined with rheumatoid arthritis, gout arthritis, ankylosing spondylitis, and Charcot’s arthritis. (2) Combined with congenital dislocation of hip joint, congenital varus foot, dwarfism, and scoliosis.

### Study design and observation indicators

This study was a retrospective case-control study. All patients had the full right to be informed and were approved by the hospital ethics committee. The full length weight-bearing radiography of both lower limbs which was taken with internal rotation of both lower limbs at 15°, plain CT scan, and MRI examination of knee joint was completed in all patients upon admission. The Miniaci method was used for preoperative planning, and the target weight-bearing line ranging from 55 to 62.5% was set according to the patient’s symptoms and the degree of osteoarthritis. Full-length weight-bearing radiographs were reexamined at the 1-year follow-up and changes in tibia length, lower limbs length, FTA on the operative side, and LLD were measured before and 1 year after surgery. The length of the tibia was defined as the distance from the midpoint of the tibial plateau to the distal center of the tibia, while the length of the lower limbs was the functional whole leg length defined as the distance from the top of the femoral head perpendicular to the floor [9]. The changes in tibia length and lower limb length were the difference of postoperative length minus preoperative length. LLD was defined as the length on the operated side minus that on the healthy side.

One year after the operation, the patient’s subjective discomfort due to unequal length of lower limbs was recorded as symptomatic LLD, which was divided into “occasionally” and “frequently” according to the frequency of attacks. Less than once a week was defined as occasional, the other as frequent. The KOOS score subcategories of pain, symptoms, activities of daily living (ADL), sports/recreations, and quality of life (QOL) were used to evaluate patients’ clinical outcomes. Patients’ age, gender, and BMI were recorded simultaneously at 1 year after surgery, and the association between these factors and symptomatic LLD was analyzed together with degrees of LLD to screen for high-risk factors.

### Surgical procedures and rehabilitation

All patients were treated with a medial tibial distraction osteotomy to correct varus deformity. After routinely disinfecting the operative region and exposure, two Kirschner wires were implanted approximately 3–4 cm below the medial aspect of the tibial plateau, pointing to the capitulum fibulae. Then, the position was adjusted to be appropriate under fluoroscopy. Biplanar osteotomy was performed under the guidance of parameters such as correction angle and open height obtained by Miniaci preoperative planning. The wedge gap was opened subsequently, and a metal rod was used to evaluate the position of lower limb weight-bearing line under fluoroscopy. When the intersection of metal rod and tibial plateau located near Fujisawa point, Tomofix was used for fixation, and allograft bone was implanted into the wedge gap. Then, the incision was aseptically closed and a negative pressure drainage tube was placed. Patients were walking with the aid of a walker and partially weight bearing 2 days post-operation. Patients regained full weight bearing 6 weeks post-operation.

### Statistical analysis

Statistical analysis was performed using SPSS 25.0. Age, tibia length, lower limb length, LLD, and BMI were all measurement data, which were continuous variables. The statistical results were expressed as (‾X ± S), and the paired t test was used for statistical analysis. Gender, incidence of symptomatic LLD, etc., were used as counting data. Chi-square test or Fisher’s exact probability method were used for statistical analysis. *P* < 0.05 indicated that the difference was statistically significant.

Binary logistic regression analysis was used to analyze the risk factors of symptomatic LLD, and the regression method was forward: LR. Symptomatic LLD was the dependent variable and was assigned as 1, while asymptomatic LLD was 0. Age, gender, BMI, and degree of LLD were the covariates. Age < 55 years old was assigned as 1 and age ≥ 55 years old was assigned as 2. Male patients was assigned as 1, female patients was assigned as 0. BMI < 28.0 was assigned as 1, BMI ≥ 28 was assigned as 2. LLD < 10 mm was assigned as 1, and ≥ 10 mm was assigned as 2.

## Results

### Demographic characteristics

Of the 64 patients, 37 were males and 27 were females, 29 were aged ≥ 55 years, 35 were aged < 55 years mean age of all were 54.3 ± 7.3 years,19 were BMI ≥ 28 patients, 45 were aged < 28 patients, mean BMI (26.6 ± 2.8), 55 were in K-L grade III and 9 were in K-L grade II. There were 35 left knee and 29 right knee receiving operation. There were 18 patients with subjective LLD uncomfortableness, 13 of them were occasional and 5 were frequent. All of them stated that the symptoms were tolerable and did not negatively affect their lives (Tables 1 and 3).

**Table 1:** Demographics of patients

Item	Parameters
Gender, male, female	37,27
Age, ≥ 55, < 55, average	29,35,54.3 ± 7.3
BMI, ≥ 28, < 28, average	19,45,26.6 ± 2.8
Postoperative LLD, ≥ 10 mm, < 10 mm, average	20,44,7.7 ± 3.7
Operation side, right/left, No. of knees	29,35
K-L grades, III, II	55,9
Symptomatic LLD, yes, no	18,46

### Changes of radiography parameters and clinical scores

Patients had a mean correction angle of 11.7° ± 4.6°, with a mean increase in tibial length of 6.0 ± 3.5 mm, lower limb length of 7.5 ± 2.3 mm, and LLD of 6.9 ± 4.2 mm at 1 year post-operation. Preoperative and postoperative changes in tibia length and lower limb length were statistically significant (*P* < 0.0001).There were statistically significant differences in pain, symptoms, ADL, sports/recreations, QOL of KOOS subclassification before and after surgery (*P* < 0.0001) (Table 2).

**Table 2:** Comparison of preoperative and postoperative radiography parameters and clinical outcomes

	Pre-op	1 year post-op	Change	*P*
Tibial length	344.7 ± 19.1	350.7 ± 19.0	6.0 ± 3.5	< 0.0001
Lower limb length	842.4 ± 38.3	849.8 ± 38.4	7.5 ± 2.3	< 0.0001
Limb length discrepancy	− 6.9 ± 3.5	6.9 ± 4.2	13.7 ± 4.8	< 0.0001
FTA	172.6 ± 2.4	184.3 ± 3.2	11.7 ± 4.6	< 0.0001
KOOS pain	33.0 ± 6.3	74.5 ± 6.8		< 0.0001
KOOS symptoms	45.1 ± 8.6	71.5 ± 10.1		< 0.0001
KOOS ADL	41.5 ± 8.7	76.7 ± 10.9		< 0.0001
KOOS Sport/Rec	36.9 ± 9.4	63.0 ± 8.0		< 0.0001
KOOS QOL	35.8 ± 7.9	61.5 ± 5.9		< 0.0001

*Pre-op* pre-operation, *post-op* post-operation, *FTA* femorotibial angle

### Screening and regression analysis of risk factors for symptomatic LLD

Gender, age, BMI, and degree of LLD may be high-risk factors for symptomatic LLD as determined by prior statistical screening and were included in the regression analysis model. Binary logistic regression revealed that age ≥ 55, BMI ≥ 28, and LLD ≥ 10 mm were high-risk factors for symptomatic LLD (*P* = 0.031, OR = 4.82; *P* = 0.012, OR = 6.251; *P* = 0.006, OR = 6.836) (Tables 3, 4, and 5).

**Table 3:** Information for patients with symptomatic LLD after OWHTO

Patient numbers	Gender (M = 1, F = 0)	Age(years)	BMI (kg/m^**2**^)	Change in tibial length	Change in lower limb length	Limb length discrepancy	Correction angle	Frequencies
1	1	57	32.5	6.5	7.3	10.6	17.4	Frequently
2	0	65	22.4	10.3	5.0	6.7	10.1	Occasionally
3	0	54	20.5	2.6	10.2	12.7	16.8	Occasionally
4	0	57	28.4	5.4	8.8	2.0	16.5	Occasionally
5	1	50	29.2	9.7	10.7	3.6	11.1	Occasionally
6	0	59	27.8	9.3	10.3	5.1	19.1	Frequently
7	1	44	29.7	3.1	11.1	14.8	19.7	Occasionally
8	0	63	35.6	3.2	7.1	7.5	17.3	Occasionally
9	1	56	26.1	8.4	6.7	2.2	7.9	Occasionally
10	0	54	30.2	1.8	6.7	13.9	11.0	Occasionally
11	1	42	28.9	0.6	8.9	12.5	8.5	Occasionally
12	1	60	27.5	8.1	4.4	5.4	12.2	Frequently
13	0	56	22.9	6.9	4.2	11.2	11.4	Occasionally
14	0	64	28.9	3.4	3.7	9.5	12.5	Occasionally
15	0	58	26.4	1.5	7.1	13.2	11.9	Frequently
16	1	59	26.2	10.3	5.2	12.3	16.4	Frequently
17	1	57	25.0	5.7	10.0	-2.7	12.8	Occasionally
18	1	41	28.7	5.4	12.0	14.0	12.2	Occasionally

**Table 4:** Screening for high-risk factors of symptomatic LLD

	Symptomatic LLD	Asymptomatic LLD	*P*
**Gender**			
Male	10	27	0.05
Female	8	19	
**Age**			
≥ 55	12	17	0.03
< 55	6	29	
**BMI**			
≥ 28	9	10	0.03
< 28	9	36	
**LLD**			
≥ 10 mm	9	6	< 0.01
< 10	9	40

**Table 5:** Binary logistic analysis of risk factors for symptomatic LLD

	B	SE	Wald	***P***	OR	95%OR
**Age**	1.573	0.727	4.679	0.031	4.82	1.2–20.0
**BMI**	1.833	0.732	6.276	0.012	6.251	1.5–26.2
**LLD**	1.922	0.704	7.451	0.006	6.836	1.7–27.2
**Constant**	− 8.362	2.206	14.367	0.000	0.000	

*B* regression line coefficient, *SE* standard error, *OR* odds ratio

## Discussion

The main findings of this study were that patients with age ≥ 55, BMI ≥ 28, and postoperative LLD ≥ 10 mm had a higher probability of developing symptomatic LLD after OWHTO.

For osteoarthritis patients with varus deformity of the lower limbs, due to the influence of space narrowing and varus angulation, the effective length of lower limbs tends to be shortened. After osteotomy or orthodontic surgery, the length of lower limbs is also prone to change due to the opening and closing of the bony structure, among which, medial open osteotomy is more likely to lead to lengthening of the limbs [2, 5, 10]. Mathematical models had predicted that correction of 10° deformity in OWHTO would result in an extension of lower limb length of 17–20.5 mm, while in actual studies the change of lower limb length was less than in the theoretical model [5, 11]. Bae DK et al. found an average extension of 6.2–7.8 mm in lower limbs after OWHTO on both intraoperative computer-based navigation and radiographic measurements, and there was only one patient with extension of more than 10 mm [5]. In another study, Kim Ji et al. also demonstrated an average 7.6 mm lengthening of lower limbs after OWHTO and found that the lengthening was positively correlated with the open height of the wedge gap [6]. Our study found a mean extension of 7.5 mm in the lower limbs of postoperative patients, which was similar to the above reports. However, it should be noted that the length of lower limbs refers to the distance from the top of the femoral head to the center of the tibial plafond in literatures, whereas it refers to the distance from the top of the femoral head perpendicular to the floor in this study.

Studies have found that only 10% of the normal population has definite bilateral lower limb isometric length, and 90% of individuals present with LLD within 1 cm [8, 12]. There is a certain compensatory mechanism of the body, and not all patients with LLD have symptoms [13]. The theoretical model study found that the body could compensate for LLD by the coronal pelvic tilt and the flexion of knee and hip joint on the side of the long leg under static conditions, and the pelvic tilt was about 6.1° when the LLD reached 2–3 cm. Nonetheless, under dynamic conditions, the tilt of the pelvis decreased and the shorter lower limb was extended through the ankle plantarflexion, while the longer side was compensated by hip and knee flexion and ankle dorsal extension [14, 15]. It has been reported that when the chronic LLD is less than 2 cm, most people can tolerate it [8, 16]. However, even small LLD can bring long-term adverse effects, such as accelerated degeneration of hip and knee joints on the long side and the lumbar spine [17, 18]. It had been documented [8, 19] that acute LLD was more likely to make patients feel subjectively uncomfortable, and even patients with LLD < 5 mm may suffer LLD symptoms abidingly. The cases we present showed that patients with LLD ≥ 10 mm had a 6.84-fold higher risk of developing LLD symptoms than those with LLD < 10 mm, but not all patients with LLD ≥ 10 mm were symptomatic, indicating that the degree of LLD was an important factor in inducing symptoms, but not the only factor.

Another finding of our study was that patients with BMI ≥ 28 were more likely to have symptoms of LLD. Biomechanics of the lumbar spine, pelvis, and lower limbs were altered after acute LLD, which was more significant in patients with greater BMI. The compensation of the body may lead to the stress concentration of a joint, a muscle, or ligament, and that may induce uncomfortable symptoms over time, especially for patients with BMI ≥ 28. A study [7] found that lower back pain was more likely to occur among meat cutters and standing service workers than office staff for patients, which indirectly confirmed the inductive effect of body weight on symptomatic LLD. However, in patients aged ≥ 55, who tend to already have some degree of degeneration in the hip, knee, and lumbar spine, as well as declining the muscle and ligament function due to their older age, and thus less tolerance to acute LLD after OWHTO, resulting in a higher risk of developing LLD symptoms. A study of predictors of self-perceived LLD after total hip arthroplasty has identified BMI < 26 and an increase in LLD of more than 5 mm was the high-risk factor [20].

This study has some limitations. Firstly, this study is a retrospective study with a small sample size and a lack of CWHTO cases as the control group. Secondly, in this study, the length was measured on the full-length radiography of both lower limbs in the weight-bearing position, and the average length was obtained after the independent measurement by two researchers. However, some measurement errors may exist due to the influence of radiation angle and distance. Thirdly, in the current literature, the symptoms of LLD were mostly evaluated by low back pain, abnormal gait, and subjective feelings. In this group of cases, the LLD was relatively small, and questionnaire was used to ask whether the patients had the uncomfortableness of LLD which can be used to identify the symptoms, but the degree cannot be evaluated. In the future, it still needs to be confirmed by prospective studies with larger sample size.

## Conclusion

Age, BMI, and degree of LLD are high-risk factors for symptomatic LLD. Patients with age ≥ 55, BMI ≥ 28, and postoperative LLD ≥ 10 mm are more likely to develop symptomatic LLD. Older or heavier patients, who are expected to have an LLD greater than 10 mm after OWHTO, should be fully informed of the possibility of postoperative LLD symptoms.

## Data Availability

The datasets during and/or analyzed during the current study available from the corresponding author on reasonable request.

## Abbreviations

OA: Osteoarthritis
OWHTO: Open wedge high tibial osteotomy
CWHTO: Closed wedge high tibial osteotomy
FTA: Femorotibial angle
LDFA: Lateral distal femoral angle
MPTA: Medial proximal tibia angle
LLD: Lower limb discrepancy
LCL: Lateral collateral ligament
MCL: Medial collateral ligament
